# Generation of multipartite entanglement between spin-1 particles with bifurcation-based quantum annealing

**DOI:** 10.1038/s41598-022-17621-1

**Published:** 2022-09-02

**Authors:** Yuichiro Matsuzaki, Takashi Imoto, Yuki Susa

**Affiliations:** 1 Research Center for Emerging Computing Technologies, National Institute of Advanced Industrial Science and Technology (AIST), 1-1-1 Umezono, Tsukuba, Ibaraki 305-8568 Japan; 2 NEC-AIST Quantum Technology Cooperative Research Laboratory, National Institute of Advanced Industrial Science and Technology (AIST), Tsukuba, Ibaraki 305-8568 Japan; 3 System Platform Research Laboratories, NEC Corporation, Kawasaki, Kanagawa 211-8666 Japan

**Keywords:** Quantum information, Quantum simulation

## Abstract

Quantum annealing is a way to solve a combinational optimization problem where quantum fluctuation is induced by transverse fields. Recently, a bifurcation-based quantum annealing with spin-1 particles was suggested as another mechanism to implement the quantum annealing. In the bifurcation-based quantum annealing, each spin is initially prepared in $|0\rangle$, let this state evolve by a time-dependent Hamiltonian in an adiabatic way, and we find a state spanned by $|\pm 1\rangle$ at the end of the evolution. Here, we propose a scheme to generate multipartite entanglement, namely GHZ states, between spin-1 particles by using the bifurcation-based quantum annealing. We gradually decrease the detuning of the spin-1 particles while we adiabatically change the amplitude of the external driving fields. Due to the dipole-dipole interactions between the spin-1 particles, we can prepare the GHZ state after performing this protocol. We discuss possible implementations of our scheme by using nitrogen vacancy centers in diamond.

## Introduction

Quantum annealing (QA) is a technique for solving combinational optimization problems^1–3^. The solution of the combinational optimization problems is embedded in the ground state of the Ising Hamiltonian^4^, which is called the problem (or target) Hamiltonian. We use the transverse magnetic fields to induce quantum fluctuation, and this Hamiltonian is called the driving Hamiltonian. After preparing a ground state of the driving Hamiltonian, we gradually decrease the amplitude of the transverse driving fields while we slowly increase the strength of the Ising Hamiltonian. If the dynamics is adiabatic, the ground state of the problem Hamiltonian can be prepared^5^. Previous studies mainly focus on the use of two-level systems for QA^6–9^.

The other mechanisms using bifurcation were proposed to induce the quantum fluctuations for QA. It is known that a parametrically driven Kerr nonlinear oscillator (KPO) shows the bifurcation^10^. A quantum superposition of two distinct states of the KPO can be generated by using quantum adiabatic evolution through its bifurcation point. Moreover, we can use this system as a qubit for a gate type-quantum computer^11^. Previous researches reveal that we can use the KPO for QA to find a ground state of Ising Hamiltonians^12,13^.

Recently, Takahashi shows that we can use spin-1 particles for the bifurcation-based QA^14^. For non-interacting spin-1 systems, the initial state is $|0\rangle$, and degenerate states $|\pm 1\rangle$ are prepared at the end of the evolution, which is similar to the bifurcation mechanism of the KPO. On the other hand, for interacting spin-1 systems, the problem Hamiltonian is encoded in a subspace spanned by $|\pm 1\rangle$. Each spin-1 particle is initially prepared in $|0\rangle$, and adiabatic changes of the Hamiltonian including the coupling between the spin-1 particles provide a ground state of the problem Hamiltonian^14^.

Here, we propose a scheme to generate the GHZ states between spin-1 particles by using the bifurcation-based QA. Suppose that there are dipole-dipole interactions between the spin-1 particles. By choosing suitable parameters, the GHZ states have the lowest energy. This means that, starting from a trivial ground state of $|00\cdots 0\rangle$ with longitudinal fields, we adiabatically change the Hamiltonian, and we can obtain the GHZ states where we add external transversal fields in the middle of the dynamics. Importantly, due to the degeneracy of the ground states of the target Hamiltonian, the energy gap between the ground state and excited states becomes small during QA. However, we show that the total Hamiltonian commutes with a parity operator, and this symmetry can suppress the non-adiabatic transitions during QA. Although this kind of the symmetry protected mechanism was discussed in the conventional QA^15–19^, we firstly utilize the symmetry protected mechanism for the bifurcation-based QA. Moreover, as a possible implementation, we discuss the use of nitrogen vacancy (NV) centers in diamond, and they are spin-1 particles that are candidates to realize quantum information processing.

The paper is structured as follow. In “Quantum annealing” section, we review the conventional QA and bifurcation-based QA to find a ground state of the Ising Hamiltonian. In “The nitrogen vacancy centers in diamond” section, we review the NV ceners in diamond. In “Generation of the GHZ states with bifurcation-based quantum annealing” section, we introduce our scheme to generate the GHZ states with the bifurcation-based QA. In “Numerical simulations to generate GHZ states with bifurcation-based quantum annealing” section, we perform numerical simulations to evaluate the performance of our scheme. In “Conclusion” section, we summarize our results.

## Quantum annealing

### Conventional quantum annealing with spin-1/2 particles

Here, we review the conventional QA with spin-1/2 particles^1–3^. The main aim of QA is to prepare a ground state of the following Ising-type Hamiltonian.

1 $$\begin{aligned} H^{(1/2)}_\mathrm{{P}}=\sum _{j=1}^L h_j \hat{\sigma }_z^{(j)}+ \sum _{ i\ne j} J_{i,j}\hat{\sigma }_z^{(i)}\hat{\sigma }_z^{(j)} \end{aligned}$$

where *L* denotes the number of spins, $h_j$ denotes a longitudinal field at the *j*-th spin, and $J_{i,j}$ denotes the coupling strength between the *i*-th spin and *j*-th spin. We also use a driver Hamiltonian to induce the quantum fluctuation as follows.

2 $$\begin{aligned} H^{(1/2)}_\mathrm{{D}}=\sum _{j=1}^L B_j \hat{\sigma }_x^{(j)} \end{aligned}$$

where $B_j$ denotes transverse fields. The total Hamiltonian is described as follows.

3 $$\begin{aligned} H^{(1/2)}=(1-t/T)H^{(1/2)}_\mathrm{{D}} + (t/T)H^{(1/2)}_\mathrm{{P}} \end{aligned}$$

where *T* denotes the time to implement QA. In QA, we prepare a ground state of $H_\mathrm{{D}}$, and let this state evolve by the total Hamiltonian. It is known that, as long as an adiabatic condition is satisfied, we can obtain a ground state of the total Hamiltonian.

### Bifurcation-based quantum annealing with spin-1 particles

Let us review a bifurcation-based quantum annealing with spin-1 particles^14^. We consider the following driving Hamiltonian

4 $$\begin{aligned} H_\mathrm{{D}}=\sum _{j=1}^L A(t) \hat{S}_x^{(j)}+ C(t) (\hat{S}_z^{(j)})^2 \end{aligned}$$

where $\hat{S}_x= |B\rangle \langle 0| +|0\rangle \langle B|$, $\hat{S}_y= -i|D\rangle \langle 0| +i|0\rangle \langle D|$, $\hat{S}_z=|1\rangle \langle 1|- |-1\rangle \langle -1|$, and $|B\rangle =\frac{1}{\sqrt{2}}(|+1\rangle +|-1\rangle )$, $|D\rangle =\frac{1}{\sqrt{2}}(|+1\rangle -|-1\rangle )$. We slowly change *C*(*t*) from a positive large value to a negative large value while *A*(*t*) has a finite but a small value in the middle of QA. The problem Hamiltonian is given as

5 $$\begin{aligned} H_\mathrm{{P}}=\sum _{j=1}^L h_j \hat{S}_z^{(j)}+ \sum _{i\ne j}J_{i,j}\hat{S}_z^{(i)}\hat{S}_z^{(j)} \end{aligned}$$

and the total Hamiltonian is given as

6 $$\begin{aligned} H=H_\mathrm{{D}}+H_\mathrm{{P}}. \end{aligned}$$

We set $|C(0)|=|C(T)|\gg |h_j|, |J_{i,j}|$, and the ground state of the total Hamiltonian at $t=0$ is $\bigotimes _{j=1}^L|0\rangle _j$. By letting this state evolve by the total Hamiltonian, we obtain the ground state of the problem Hamiltonian as long as the adiabatic condition is satisfied.

## The nitrogen vacancy centers in diamond

We review the Hamiltonian of the NV centers in diamond. The NV center is a spin-1 patricle, and there is a dipole-dipole interaction between the NV centers. The Hamiltonian is described as follows

7 $$\begin{aligned}H^{(\mathrm {NV})}=\sum _{j=1}^L\Big ( D^{(j)}_0(\hat{S}_z^{(j)})^2 + E_x^{(j)}( (\hat{S}_x^{(j)})^2 - (\hat{S}_y^{(j)})^2 ) \Big ) +\Big ( \sum _{j\ne k}J_{j,k}(\hat{S}_x^{(j)}\hat{S}_x^{(k)} +\hat{S}_y^{(j)}\hat{S}_y^{(k)} )- J' _{j,k}\hat{S}_z^{(j)} \hat{S}_z^{(k)} \Big ) . \end{aligned}$$

where $D^{(j)}_0$ denotes a zero-field splitting at the *j*-th spin, $E_x^{(j)}$ denotes a strain at the *j*-th spin, $J_{j,k}$ denotes the flip-flop interaction between the *j*-th spin and *k*-th spin, and $J'_{j,k}$ denotes the Ising interaction between the *j*-th spin and *k*-th spin. It is worth mentining that we can change the values of $D^{(j)}_0$ ($E_x^{(j)}$) by changing the temperature (amplitude of the applying electric fields)^20–24^.

The NV center is a promising candidate to realize quantum information processing. The NV center can be coupled with magnetic fields, electric fields, and temperature, and pressure^22,25,26^. We can polarize the NV centers by illuminating a green laser, and also we can readout the spin state by using the photoluminescence from the NV centers^25–27^. Moreover, the NV center has a long coherence time such as a few milliseconds^28–30^. The NV center can be coherently coupled with an optical photon^31^. These properties are prerequisite for the NV centers to be candidates for the quantum sensing^25,32–34^, a quantum memory for a superconducting qubit^35–38^, quantum communication^39^, and distributed quantum computation^40^. Especially, NV centers could be used to realize an entanglement-enhanced quantum sensing with the GHZ states^41–48^ or could be used for a quantum network with encoding where the GHZ states are resource to construct an error correcting code^49–51^.

## Generation of the GHZ states with bifurcation-based quantum annealing

**Figure 1:**
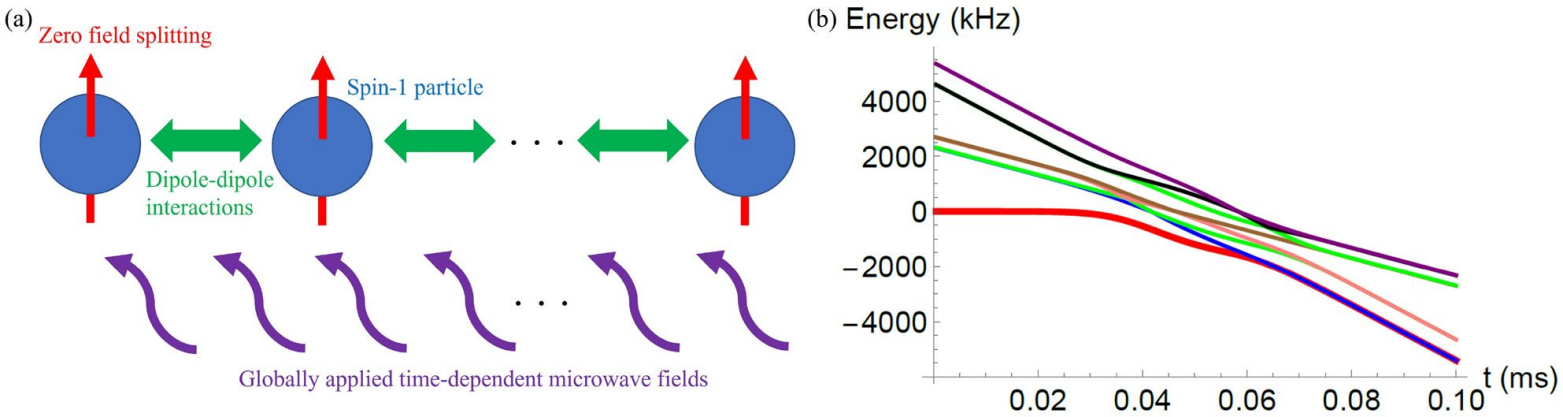
(**a**) Schematic of our proposal. Spin-1 particles with zero-field splitting are arranged in a one-dimensional chain. There are dipole-dipole interactions between the spin-1 particles. We globally apply time-independent microwave fields to implement the bifurcation-based quantum annealing. (**b**) We plot eigenenergies against the time with the unit of milliseconds where we adopt the Hamiltonian in the Eq.(9), which is derived by using a rotating wave approximation. The energy gap between the ground state and first excited state becomes smaller as *t* approaches to *T*. We set the parameters as $L=2$, $D_0'/2\pi = 400$ kHz, $E_x^{(1)}/2\pi = 1$ kHz, $E_x^{(1)}/2\pi = 1.2$ kHz, $J_{12}/2\pi =30$ kHz, $J_{12}'/2\pi =60$ kHz, $B/2\pi =100$ kHz, $\sigma =0.2 T$, $T=0.1$ ms.

We explain our scheme to generate a GHZ state between spin-1 particles with the bifurcation-based QA. The GHZ states are defined as $|\mathrm{{GHZ}}_{\pm } \rangle =\frac{1}{\sqrt{2}}\bigotimes _{j=1}^L|1\rangle _j\pm \frac{1}{\sqrt{2}}\bigotimes _{j=1}^L|-1\rangle _j$. The schematic is shown in Fig. 1a. We consider to apply our scheme with the NV centers in diamond. Importantly, in an experiment, it is difficult to have a negative value of $D^{(j)}_0$. Although we can slightly change the value of $D^{(j)}_0$ by changing the temperature, the value of $D^{(j)}_0$ is as large as $2\pi \times 2.88$ GHZ, and there is no experiment to change the value of the zero field splitting to the negative values, which requires a frequency shift of a few GHZ. To overcome this problem, we adopt an idea of a spin-lock QA where the system driven by microwave fields is in a rotating frame^52–54^. The advantage of this scheme is that the detuning between the resonant frequency of the spins and the microwave frequency plays an role of the longitudinal fields, and we can easily set the negative detuning by setting a suitable value of the microwave frequency. When the NV centers are arranged in a one dimensional chain and microwave driving field are applied along *x* direction, the Hamiltonian is described as follows.

8 $$\begin{aligned}H=\sum _{j=1}^L\Big ( D^{(j)}_0(\hat{S}_z^{(j)})^2 +2 \lambda ^{(j)}_x (t) \cos \omega t\ \hat{S}_x ^{(j)} + E_x^{(j)}( (\hat{S}_x^{(j)})^2 - (\hat{S}_y^{(j)})^2 ) \Big ) +\Big ( \sum _{j\ne k}J_{j,k}(\hat{S}_x^{(j)}\hat{S}_x^{(k)} +\hat{S}_y^{(j)}\hat{S}_y^{(k)} )- J' _{j,k}\hat{S}_z^{(j)} \hat{S}_z^{(k)} \Big ) \end{aligned}$$

where $\lambda ^{(j)}_x (t)$ ($\omega$) denotes the amplitude (frequency) of the microwave driving at the *j*-th NV center. The dipole-dipole interactions decrease by $1/r^3$ where $r=|j-k|$ denotes the distance between the spins. For example, $J_{13}=\frac{1}{8}J_{12}$ is satisfied. In a rotating frame defined by $U={\rm{Exp}}[-i \sum _{j=1}^{L}\omega t (\hat{S}_z^{(j)}) ]$, we obtain

9 $$\begin{aligned} H & \simeq \sum\limits_{{j = 1}}^{L} ( D^{\prime}_{j} (\hat{S}_{z}^{{(j)}} )^{2} + \lambda _{x}^{{(j)}} (t)\hat{S}_{x}^{{(j)}} + E_{x}^{{(j)}} ((\hat{S}_{x}^{{(j)}} )^{2} - (\hat{S}_{y}^{{(j)}} )^{2} )) \\ & \quad + (\sum\limits_{{j \ne k}} {J_{{j,k}} } (|B\rangle _{j} \langle 0| \otimes |0\rangle _{k} \langle B| + |D\rangle _{j} \langle 0| \otimes |0\rangle _{k} \langle D| + {\text{hc}}) - J^{\prime}_{j,k} \hat{S}_{z}^{{(j)}} \hat{S}_{z}^{{(k)}} ) \\ \end{aligned}$$

where we define $D'\equiv D_0 -\omega$ and we use a rotating wave approximation (RWA). In the real experiments, we can easily change the frequency of the microwave driving while the dynamical control of the zero-field splitting is difficult. So we assume that $D_0$ is constant while we change $\omega$ during QA. Throughout of our paper, we set the following.

10 $$\begin{aligned} D'= & {} -D'_0\tanh [M(t-\frac{T}{2})/T] \end{aligned}$$

11 $$\begin{aligned} \lambda _x(t)= & {} B e^{-(t-\frac{T}{2})^2/\sigma ^2} \end{aligned}$$

where we set $M=5$. At $t=0$ ($t=T$), the ground state of the Hamiltonian after the RWA is approximately described by $|\psi _0\rangle =\bigotimes _{j=1}^L|0\rangle _j$ for $\sigma \gg T$. On the other hand, at $t=T$, degenerate ground states of the Hamiltonian after the RWA are described by $|\mathrm{{GHZ}}_{\pm } \rangle =\frac{1}{\sqrt{2}}\bigotimes _{j=1}^L|1\rangle _j\pm \frac{1}{\sqrt{2}}\bigotimes _{j=1}^L|-1\rangle _j$ for $\lambda _x(t)=0$ and $E_x= 0$. We plot an energy diagram of the Hamiltonian (9) in Fig. 1b, and we confirm that the energy gap between the ground state and first excited state becomes smaller as the time *t* approaches to *T*.

Importantly, the Hamiltonian in the Eq. (9) commutes with a parity operator of $\hat{P}=\bigotimes _{j=1}^L ( |B\rangle _j\langle B| -|D\rangle _j\langle D| +|0\rangle _j\langle 0| )$, and we have $\hat{P}|\psi _0\rangle =|\psi _0\rangle$ while we have $\hat{P}|\mathrm{{GHZ}}_{\pm } \rangle = (\pm 1 )|\mathrm{{GHZ}}_{\pm }\rangle$. Therefore, by preparing a state of $|\psi _0\rangle$, the adiabatic change in the Hamiltonian allows us to create the state of $|\mathrm{{GHZ}}_{+} \rangle$ where non-adiabatic transitions between $|\mathrm{{GHZ}}_{+} \rangle$ and $|\mathrm{{GHZ}}_{-} \rangle$ are prohibited due to the difference of the symmetry.

## Numerical simulations to generate GHZ states with bifurcation-based quantum annealing

To evaluate the performance of our scheme, we perform numerical simulations to plot the fidelity between the target GHZ state and the state after QA. Here, we adopt the Hamiltonian in the Eq. (8). To consider the decoherence, we use the following GKSL master equation^55,56^

12 $$\begin{aligned} \frac{d\rho }{dt}=-i[H,\rho ] +\sum _{j=1}^{L}\frac{\gamma }{2} (2\hat{L}_j \rho \hat{L}_j^{\dagger }-\hat{L}^{\dagger }_j \hat{L}_j \rho -\rho \hat{L}^{\dagger }_j \hat{L}_j )\ \end{aligned}$$

where $\gamma$ denotes a decoherence rate and $\hat{L}_j$ denotes a lindblad operator at the *j*-site. Throughout of this paper, we use $\hat{L}_j=\hat{S}_z^{(j)}$, which corresponds to magnetic field noise that is typical for the NV centers^57–60^. We define a fidelity as $F=\langle \mathrm{{GHZ}}_+|\rho (t)|\mathrm{{GHZ}}_+ \rangle$.

**Figure 2:**
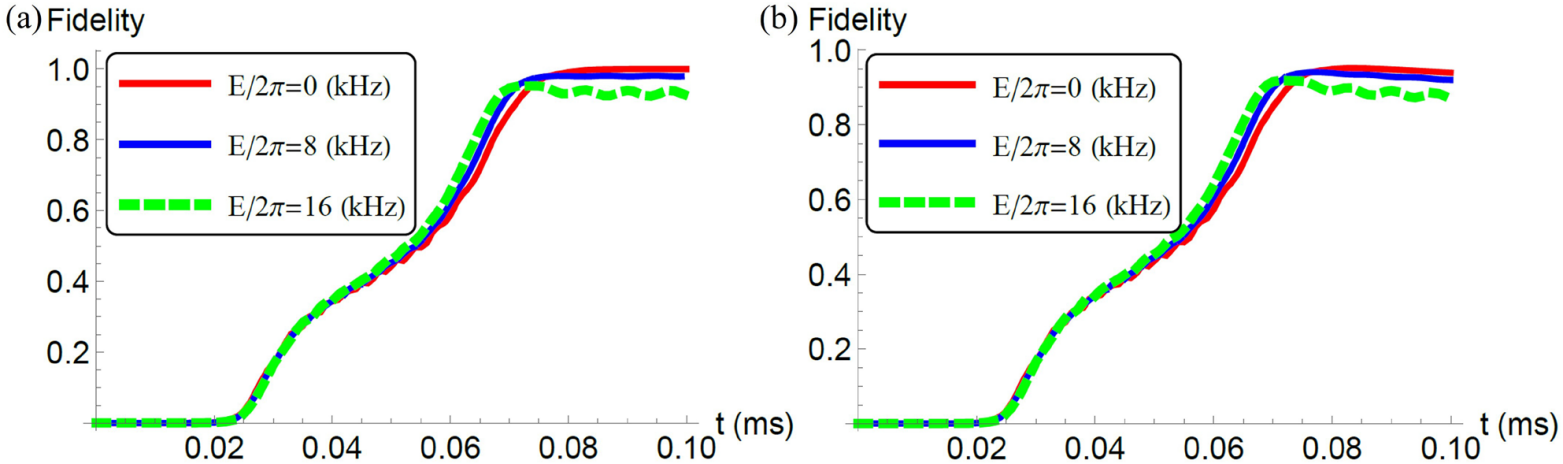
(**a**) We plot a fidelity against the time with the unit of milliseconds where we adopt the Hamiltonian without a rotating wave approximation. We set the parameters as $L=2,$ $D_0'/2\pi = 200$ kHz, $E_x^{(1)}/2\pi = E$ kHz, $E_x^{(2)}/E_x^{(1)}= 1.2$, $J_{12}/2\pi =30$ kHz, $J_{12}'/2\pi =60$ kHz, $B/2\pi =340$ kHz, $\sigma =0.2 T$, $T=0.1$ ms, $\gamma =0$, and $\omega =40$ MHz. (**b**) We plot a fidelity against the time under the effect of decoherence with $\gamma =0.5$ kHz. We use the same parameters as that used in (**a**) except the decoherence rate.

We plot the fidelities against *t* for $L=2$ without decoherence in Fig. 2a. When there is no strain, the fidelity is more than 0.999, and this means that the adiabatic condition is reasonably satisfied. When we add the effect of the strain, the fidelity becomes as small as 0.979 (0.925) for $E/2\pi =8$ ($E/2\pi =16$) kHz, as shown in Fig. 2a. This comes from the fact that a ground state of the Hamiltonian with the strain is not the GHZ state. To obtain a high-fidelity GHZ state among the NV centers, it is crucial to suppress the effect of the strain by applying suitable amount of the electric fields. In the real experiment, we have $D_0/2\pi \simeq \omega /2 \pi \simeq 2.88$ GHz. However, the computational cost becomes expensive when $D_0/2\pi$ is much larger than the other parameters. Therefore, throughout of this paper, we set $D_0/2\pi \simeq \omega /2 \pi = 40$ MHz. Since we confirm that the dynamics does not significantly change even when we increase $D_0/2\pi$ and $\omega /2\pi$ around this parameter range, we believe that our numerical simulations are still useful to predict the experimental results for $D_0/2\pi \simeq \omega /2 \pi \simeq 2.88$ GHz.

Also, we plot the fidelity under the effect of decoherence against *t* for $L=2$ in Fig. 2b. Compared with the fidelity by using the unitary dynamics plotted in Fig. 2a, the fidelity becomes smaller as expected. However, the fidelity is still around 0.9, and so these results show that we can generate the GHZ states even under noisy environments.

Importantly, there was an experimental demonstration to generate an entanglement between two NV centers^61^. However, the previous scheme requires a complicated pulse sequence, and the necessary number of the pulse operations increases as the number of NV centers increases. Moreover, the NV centers should be individually controlled by using frequency selectivity. On the other hand, our protocol just requires global applications of the microwave pulses without individual adressing of the NV centers, which would be beneficial to generate a GHZ states with more than two NV centers.

**Figure 3:**
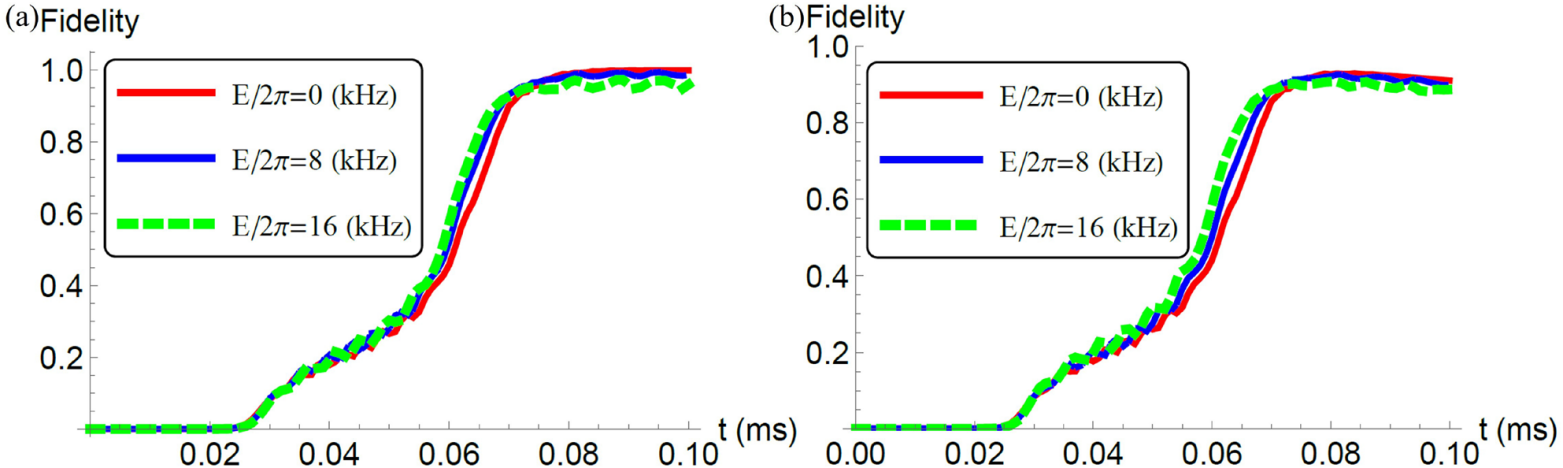
(**a**) We plot a fidelity against the time with the unit of milliseconds where we adopt the Hamiltonian without a rotating wave approximation. We set the parameters as $L=3,$ $D_0'/2\pi = 200$ kHz, $E_x^{(1)}/2\pi = E$ kHz, $E_x^{(2)}/E_x^{(1)}= 1.2$, $E_x^{(3)}/E_x^{(2)}= 1.2$, $J_{12}/2\pi =J_{23}/2\pi =30$ kHz, $J_{12}'/2\pi =J_{23}'/2\pi =60$ kHz, $J_{12}/J_{13}=J'_{12}/J'_{13}=8$, $B/2\pi =340$ kHz, $\sigma =0.2 T$, $T=0.1$ ms, $\gamma =0$, and $\omega =40$ MHz. (**b**) We plot a fidelity against the time under the effect of decoherence with $\gamma =0.5$ kHz. We use the same parameters as that used in (**a**) except the decoherence rate.

Moreover, we plot the fidelities against *t* for $L=3$ with and without decoherence, as shown in Fig. 3a,b, respectively. When we consider the unitary dynamics, the fidelities with $L=3$ are comparable with those with $L=2$, as shown in Fig. 3a. This means that, for $L=3$, the adiabatic conditions are reasonably satisfied. With decoherence, the fidelities becomes worse than those without decoherence. However, as shown in Fig. 3b, the fidelities are still around 0.9. Again, these results show the practicality of our scheme.

**Figure 4:**
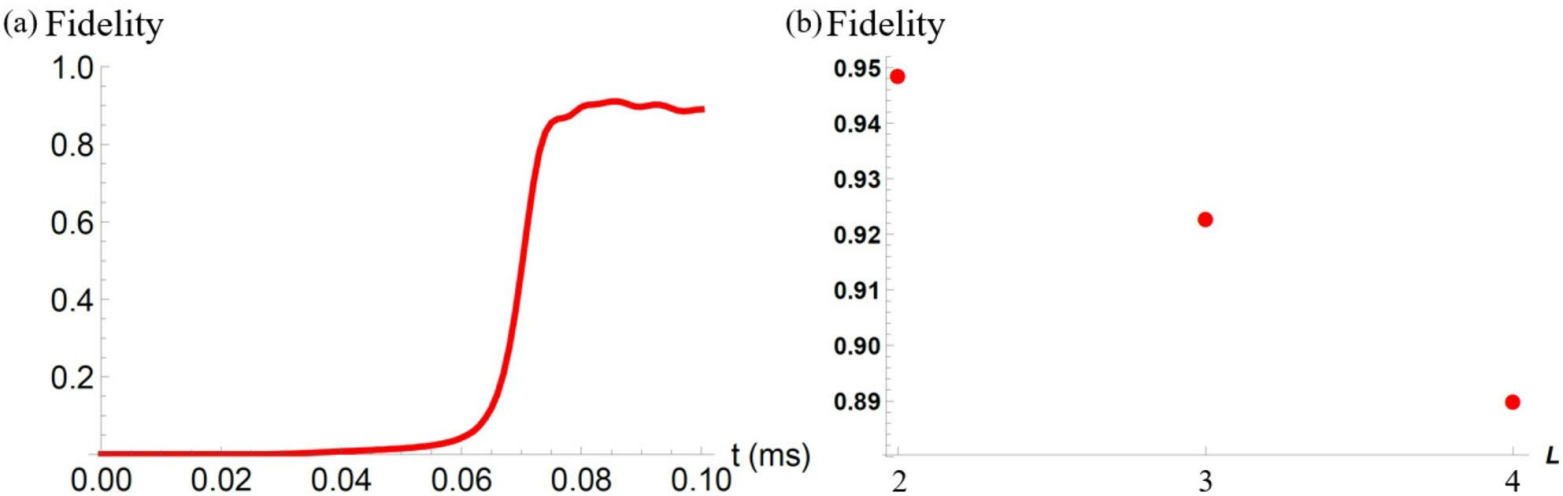
(**a**) We plot a fidelity against the time with the unit of milliseconds where we adopt the Hamiltonian with a rotating wave approximation. We set the parameters as $L=4,$ $D_0'/2\pi = 200$ kHz, $E_x^{(1)}/2\pi =E_x^{(2)}/2\pi =E_x^{(3)}/2\pi = E_x^{(4)}/2\pi =3$ kHz, $J_{12}/2\pi =J_{23}/2\pi =J_{34}/2\pi =30$ kHz, $J_{12}'/2\pi =J_{23}'/2\pi =J_{34}'/2\pi =60$ kHz, $J_{12}/J_{13}=J'_{12}/J'_{13}=J_{23}/J_{24}=J'_{23}/J'_{24}=8$, $J_{12}/J_{14}=J'_{12}/J'_{14}=27$, $B/2\pi =340$ kHz, $\sigma =0.2 T$, $T=0.1$ ms, and, $\gamma =0.5$ kHz. (**b**) We plot the fidelity agains the number of qubits where we adopt the Hamiltonian with a rotating wave approximation. We choose $E_x^{(j)}/2\pi =3$ kHz for $j=1,2,3,4$. For $L=2$ and $L=3$, we use the same parameters as that used in Figs. 2 and 3 except the strain. For $L=4$, we use the same parameters as that used in (**a**).

Finally, we plot the fidelity agaisnt the number of qubits. For this purpose, let us firstly show the fidelity with $L=4$ in Fig. 4a. To save the computational cost, we adopt the Hamiltonian with a rotating wave approximation. This result shows that the fidelity is as high as 0.89. Then, we plot the fidelity against the number of qubits in Fig. 4b. Roughly speaking, the fidelity decreases by 0.03 as we add another NV center.

## Conclusion

In conclusion, we propose a scheme to generate GHZ states between spin-1 particles by using bifurcation-based QA. Suppose that there are dipole-dipole couplings between the spin-1 particles. After each spin-1 particle is prepared in $|0\rangle$, we slowly turn on the microwave driving, and we finally turn off the the microwave driving in an adiabatic way. We show that adiabatic changes in frequency and amplitude of the microwave driving fields provide a GHZ states after QA. Although the energy gap between the ground state and first excited state becomes nearly degenerate when we turn off the microwave driving fields, we show that a symmerty of the Hamiltonian protects the state from the non-adiabatic transitions. Our scheme could be useful for possible applications to quantum information processing by using nitrogen vacancy centers in diamond. Our method could be extended to the preparation of other entangled states such as spin cat states (that are considered to be generalized GHZ states^62–64^) or W states^65^. There are four key points to implement our scheme. First, we need to design a problem Hamiltonian whose ground state is the target entangled state. Second, the Hamiltonian should commute with a symmetry operator (such as a parity operator). Third, the ground state of the driver Hamiltonian should belong to the same sector of the symmetry operator as the ground state of the problem Hamiltonian. Fourth, the first excited state of the problem Hamiltonian should belong to a different sector of the symmetry operator as the ground state of the problem Hamiltonian. If these conditions are satisfied, our method can be used to prepare other entangled states. Detailed investigation whether these conditions are satisfied for other entanglement is beyond the cope of the paper, and therefore we leave this as a future work.

The datasets generated and/or analysed during the current study are not publicly available due internal rules of the AIST but are available from the corresponding author on reasonable request.

## References

[1] Kadowaki T, Nishimori H (1998). Quantum annealing in the transverse Ising model. Phys. Rev. E.

[2] Farhi, E., Goldstone, J., Gutmann, S. & Sipser, M. Quantum computation by adiabatic evolution. arXiv preprint quant-ph/0001106 (2000).

[3] Farhi E (2001). A quantum adiabatic evolution algorithm applied to random instances of an np-complete problem. Science.

[4] Lucas A (2014). Ising formulations of many NP problems. Front. Phys..

[5] Morita S, Nishimori H (2008). Mathematical foundation of quantum annealing. J. Math. Phys..

[6] Santoro GE, Martoňák R, Tosatti E, Car R (2002). Theory of quantum annealing of an Ising spin glass. Science.

[7] Johnson MW (2011). Quantum annealing with manufactured spins. Nature.

[8] Boixo S, Albash T, Spedalieri FM, Chancellor N, Lidar DA (2013). Experimental signature of programmable quantum annealing. Nat. Commun..

[9] Boixo S (2014). Evidence for quantum annealing with more than one hundred qubits. Nat. Phys..

[10] Wielinga B, Milburn G (1993). Quantum tunneling in a Kerr medium with parametric pumping. Phys. Rev. A.

[11] Cochrane PT, Milburn GJ, Munro WJ (1999). Macroscopically distinct quantum-superposition states as a bosonic code for amplitude damping. Phys. Rev. A.

[12] Goto H (2016). Bifurcation-based adiabatic quantum computation with a nonlinear oscillator network. Sci. Rep..

[13] Puri S, Andersen CK, Grimsmo AL, Blais A (2017). Quantum annealing with all-to-all connected nonlinear oscillators. Nat. Commun..

[14] Takahashi, K. Bifurcation-based quantum annealing with nested spins. arXiv preprint arXiv:2003.13439 (2020).

[15] Xing H, Wang A, Tan Q-S, Zhang W, Yi S (2016). Heisenberg-scaled magnetometer with dipolar spin-1 condensates. Phys. Rev. A.

[16] Hatomura T, Pawłowski K (2019). Superadiabatic generation of cat states in bosonic Josephson junctions under particle losses. Phys. Rev. A.

[17] Hatomura T (2019). Suppressing nonadiabatic transitions during adiabatic generation of highly entangled states in bosonic Josephson junctions. Phys. Rev. A.

[18] Huang J, Zhuang M, Lee C (2018). Non-Gaussian precision metrology via driving through quantum phase transitions. Phys. Rev. A.

[19] Hatomura, T., Yoshinaga, A., Matsuzaki, Y. & Tatsuta, M. Symmetry-protected adiabatic quantum metrology. arXiv preprint arXiv:2104.02898 (2021).

[20] Neumann P (2013). High-precision nanoscale temperature sensing using single defects in diamond. Nano Lett..

[21] Clevenson H (2015). Broadband magnetometry and temperature sensing with a light-trapping diamond waveguide. Nat. Phys..

[22] Dolde F (2011). Electric-field sensing using single diamond spins. Nat. Phys..

[23] Kobayashi S (2020). Electrical control for extending the Ramsey spin coherence time of ion-implanted nitrogen-vacancy centers in diamond. Phys. Rev. Appl..

[24] Iwasaki T (2017). Direct nanoscale sensing of the internal electric field in operating semiconductor devices using single electron spins. ACS Nano.

[25] Degen CL, Reinhard F, Cappellaro P (2017). Quantum sensing. Rev. Mod. Phys..

[26] Barry JF (2020). Sensitivity optimization for NV-diamond magnetometry. Rev. Mod. Phys..

[27] Gruber A (1997). Scanning confocal optical microscopy and magnetic resonance on single defect centers. Science.

[28] Balasubramanian G (2009). Ultralong spin coherence time in isotopically engineered diamond. Nat. Mater..

[29] Mizuochi N (2009). Coherence of single spins coupled to a nuclear spin bath of varying density. Phys. Rev. B.

[30] Herbschleb E (2019). Ultra-long coherence times amongst room-temperature solid-state spins. Nat. Commun..

[31] Bernien H (2013). Heralded entanglement between solid-state qubits separated by three metres. Nature.

[32] Maze JR (2008). Nanoscale magnetic sensing with an individual electronic spin in diamond. Nature.

[33] Taylor J (2008). High-sensitivity diamond magnetometer with nanoscale resolution. Nat. Phys..

[34] Balasubramanian G (2008). Nanoscale imaging magnetometry with diamond spins under ambient conditions. Nature.

[35] Kubo Y (2011). Hybrid quantum circuit with a superconducting qubit coupled to a spin ensemble. Phys. Rev. Lett..

[36] Zhu X (2011). Coherent coupling of a superconducting flux qubit to an electron spin ensemble in diamond. Nature.

[37] Saito S (2013). Towards realizing a quantum memory for a superconducting qubit: Storage and retrieval of quantum states. Phys. Rev. Lett..

[38] Zhu X (2014). Observation of dark states in a superconductor diamond quantum hybrid system. Nat. Commun..

[39] Childress L, Taylor J, Sørensen AS, Lukin MD (2005). Fault-tolerant quantum repeaters with minimal physical resources and implementations based on single-photon emitters. Phys. Rev. A.

[40] Nemoto K (2014). Photonic architecture for scalable quantum information processing in diamond. Phys. Rev. X.

[41] Wineland DJ, Bollinger JJ, Itano WM, Moore F, Heinzen DJ (1992). Spin squeezing and reduced quantum noise in spectroscopy. Phys. Rev. A.

[42] Wineland DJ, Bollinger JJ, Itano WM, Heinzen D (1994). Squeezed atomic states and projection noise in spectroscopy. Phys. Rev. A.

[43] Huelga SF (1997). Improvement of frequency standards with quantum entanglement. Phys. Rev. Lett..

[44] Shaji A, Caves CM (2007). Qubit metrology and decoherence. Phys. Rev. A.

[45] Matsuzaki Y, Benjamin SC, Fitzsimons J (2011). Magnetic field sensing beyond the standard quantum limit under the effect of decoherence. Phys. Rev. A.

[46] Chin AW, Huelga SF, Plenio MB (2012). Quantum metrology in non-Markovian environments. Phys. Rev. Lett..

[47] Chaves R, Brask J, Markiewicz M, Kołodyński J, Acín A (2013). Noisy metrology beyond the standard quantum limit. Phys. Rev. Lett..

[48] Isogawa, T., Matsuzaki, Y. & Ishi-Hayase, J. Vector DC magnetic-field sensing with reference microwave field using perfectly aligned nitrogen-vacancy centers in diamond. arXiv preprint arXiv:2112.00506 (2021).

[49] Jiang L (2009). Quantum repeater with encoding. Phys. Rev. A.

[50] Satoh T, Le Gall F, Imai H (2012). Quantum network coding for quantum repeaters. Phys. Rev. A.

[51] Zwerger M, Briegel H, Dür W (2016). Measurement-based quantum communication. Appl. Phys. B.

[52] Chen H (2011). Experimental demonstration of a quantum annealing algorithm for the traveling salesman problem in a nuclear-magnetic-resonance quantum simulator. Phys. Rev. A.

[53] Matsuzaki Y, Hakoshima H, Seki Y, Kawabata S (2020). Quantum annealing with capacitive-shunted flux qubits. Jpn. J. Appl. Phys..

[54] Imoto, T., Seki, Y. & Matsuzaki, Y. Preparing ground states of the xxz model using the quantum annealing with inductively coupled superconducting flux qubits. arXiv preprint arXiv:2112.12419 (2021).

[55] Gorini V, Kossakowski A, Sudarshan ECG (1976). Completely positive dynamical semigroups of n-level systems. J. Math. Phys..

[56] Lindblad G (1976). On the generators of quantum dynamical semigroups. Commun. Math. Phys..

[57] De Lange G, Wang Z, Riste D, Dobrovitski V, Hanson R (2010). Universal dynamical decoupling of a single solid-state spin from a spin bath. Science.

[58] Matsuzaki Y (2016). Optically detected magnetic resonance of high-density ensemble of NV-centers in diamond. J. Phys. Condens. Matter.

[59] Bauch E (2020). Decoherence of ensembles of nitrogen-vacancy centers in diamond. Phys. Rev. B.

[60] Hayashi K (2020). Experimental and theoretical analysis of noise strength and environmental correlation time for ensembles of nitrogen-vacancy centers in diamond. J. Phys. Soc. Jpn..

[61] Dolde F (2013). Room-temperature entanglement between single defect spins in diamond. Nat. Phys..

[62] Xiong H-N, Ma J, Liu W-F, Wang X (2010). Quantum fisher information for superpositions of spin states. Quantum Inf. Comput..

[63] Dooley S, McCrossan F, Harland D, Everitt MJ, Spiller TP (2013). Collapse and revival and cat states with an N-spin system. Phys. Rev. A.

[64] Tanaka T (2015). Proposed robust entanglement-based magnetic field sensor beyond the standard quantum limit. Phys. Rev. Lett..

[65] Dür W, Vidal G, Cirac JI (2000). Three qubits can be entangled in two inequivalent ways. Phys. Rev. A.

